# Anæsthesia in Dental Operations

**Published:** 1869-07

**Authors:** Thos. C. Edwards


AMERICAN JOURNAL

OF

DENTAL SCIENCE.

Vol. III. THIRD SERIES--JULY, 1869. No. 3.

### ARTICLE I.

### *Anæsthesia in Dental Operations,*

By Thos. C. Edwards, D.D.S.

Anæsthesia is defined in Medical Dictionaries as being "Privation of feeling," "Loss of the sense of touch," "Insensibility," &c. But these definitions do not suit my present purpose, not being comprehensive enough. I may add though that the above definitions are better suited for public use than the one I intend giving, in fact they express the literal meaning of anæsthesia more nearly than the one I will give, but I contend that the definition does not express the full meaning of the term as at present applied. As is well known, simple and comprehensive definitions do more towards shortening wearisome details than anything else, therefore, I feel justified in adopting the definition which follows especially as it originates from good authority.

Dr. Anstie in his work on "Stimulants and Narcotics" gives a definition of the term "Narcosis," which without argument I will state applies equally to the term anæsthesia. It is as follows:—"A physiological process in which the Nervous System is deprived of its vital characteristics, through the means of a poisoned blood supply, and which tends to produce general death of the organism by means of such deprivation." This definition I think comprehends in so many words as much as I could describe otherwise in as many pages, therefore, I feel no hesitancy in adopting it. I might substantiate my position by numerous facts and arguments, but will not intrude so far upon time and patience.

The fact is, that no arguments of mine are required to prove the correctness of the definition, as in the minds of all good physiologists the definition substantiates itself: (As a matter of course I have left local anæsthesia out of the question, the action of a local anæsthetic bearing about the same relation to chloroform or ether as used in general anæsthesia, as a mustard plaster does to some internal stimulant).

The use of anæsthetics has become very common in the various branches of Surgery since their first introduction in 1846, although for some time they were regarded with suspicion and distrust; and not without some reason, for truly to look at the symptoms produced by them, one would justly conclude that some danger lurked there; and, as the use of them has long since sadly proven, they are extremely dangerous agents and only require certain conditions (which are unfortunately very obscure) to produce death. Notwithstanding all this they came largely into use, and are now used almost universally in surgical operation even down to the extraction of a tooth. It is true that the action of anæsthetics are better understood than formerly and as a consequence they are not so dangerous when in proper hands, still statistics prove the important fact that about as many die under the action of the agents as formerly, therefore something is wrong. Either great carelessness is displayed in their use or somebody is using them that is ignorant of the conditions unfavorable to their use. I adopt the latter conclusion as the former is rather detrimental to professional honor and dignity. It might be said that the latter conclusion is equally so, but I think not, for how many doses of medicine are given when the physician is in the dark as to their action upon the system. This is the point I have been aiming at. Here we have the whole Medical World, including all its branches &c., using anæsthetics as well as many other agents less dangerous, of whose real action upon the economy they are positively ignorant, save the symptoms they produce. What a cutting satire it is upon the wisdom of the physician, how humiliating the reflection that one set of these healers of mortal ills are giving medicine according to one theory to produce a given effect while another set are giving the same agents according to some other theory to produce precisely the same effects. This is all well and good to a certain extent. but there is, I think, a limit to this as well as everything else in human affairs. This thing of the right of the physician or surgeon to use anæsthetics, considering the prevailing ignorance in regard to their action, and the known danger that attends their administration is, I think, one of serious import and one that demands attention. The showman in a menagerie has the right to put his head into the lion's jaws and risk the danger of having it bitten off as occasionally happens, but none would wittingly confer upon him the right to stick anybody else's head into the lion's jaws, for the showman has only a limited conception of the forbearance of the lion under such circumstances and he is not justified in exposing anybody else to such danger. Anæsthetics are dreadful roaring lions with capacious jaws, sufficient to gulp the poor unfortunate who so rashly thrusts his head into them, when the proper conditions for deglutition or decapitation are present. An obscure lesion of the brain, heart or lungs will sometimes fulfil these conditions, and the poor, ignorant patient quietly goes on with the anæsthesia, until he gently snaps the thread of life and death steps forward and relieves him of his burden.

This is a pretty dark picture I have drawn I admit, and might be modified considerably, but it is better to view this subject in such a light than to treat it lightly, think of it lightly, use anæsthetics rashly and some day "shuffle" some of our patients "off this mortal coil." The proportion of deaths during anæsthesia is comparatively small it is true, so are the the killed and wounded in battle to those who escape unhurt, but there are numerous recorded deaths by anæsthesia which will speak for themselves.

A great many speak up for Nitrous Oxide and defy any proof by statistics or otherwise that death has ever been produced by it and so on, but Nitrous Oxide although a mild sweet little sister as compared with her more boisterous, rampant brothers Ether and Chloroform, possesses the same lurking, dangerous disposition and may be regarded with deep suspicion. She is young yet and has not given to the world a full exhibition of her powers, but mark you, she will. Look at those livid lips, that dull starving eye, those palsied limbs, the banishment of intelligence into dreamland, that fluttering pulse and stertorous breathing and say she is harmless. Nevertheless the ignorant public *will* have them even in the most trivial operations, and Surgeons, Dentists, &c., *will* use them. So it cannot be avoided and the Surgeon's conceit in his wisdom and sagacity may hob nob with his conscience to its hearts content for aught his patient knows or cares.

But I am not arguing the entire disuse of anæsthetics in the above at all, for I think that that there are circumstances under which the production of anæsthesia is not only justifiable but necessary. In fact, to look at it in one light it is a great boon and blessing to suffering humanity. The poor, miserable patient, who after weeks or months of anguish and pain is drawn upon the table for the surgeons knife, needs something to avert the cruel shock to his nervous system which necessarily follows these operations. Delicate females also sometimes derive great benefit from the use of anæsthetics during the pains of labor, but after all, I think that in this instance they are used to produce their stimulant effects and are not used as anæsthetics.

I think it advisable and even necessary in all the operations of surgery in which great shocks to the nervous system may be expected from excessive pain, loss of blood, etc., and, in fact hardly see how the surgeon could well dispense with the use of them. But I hold to the opinion that the majority of cases in minor surgery could be disposed of without the use of anæsthetics, and especially could Dentists do so. No Dentist is ever justified in giving Ether, Chloroform or

Nitrous Oxide for the purpose of extracting a few teeth. The responsibility he assumes in such cases is a very grave one, and he should look well to the consequences that he may not have cause to reproach himself afterward.

Another very important reason for the nonuse of these agents by Dentists, is that their operations are performed invariably in the sitting posture, which is very unfavorable to a safe and perfect induction of anæsthesia; also during those stages of anæsthesia in which it would be advisable to commence operating, the Dentist finds it impossible to do so, on account of the rigidity of the jaws, in this dilemma he seeks success by producing a deeper anæsthesia in which the rigidity is succeeded by one of total relaxation; in fact he marches his patient up into the jaws of death and trusts to luck for him to get out.

I could enumerate many reasons for not using anæsthetics in dental operations but consider it a useless waste of time. If it could be proven satisfactorily that Nitrous Oxide was perfectly safe, and so far it has not, I would welcome it as as gift from Nature's laboratory, and would use it with much pleasure. As to ether and chloroform they are out of the question, I do not hesitate to deprecate in the strongest terms their use by any Dentist. I feel when I am using these agents that I am dealing with agents, the actions of which I do not clearly understand; and I am doing myself no injustice in saying so, for the best physiologists have not sifted the matter perfectly. I only know that when an anæsthetic is administered it seeks the vitals, sucking up the springs of life, overwhelming all those avenues through which the mind communicates with the outer world, and finally if the anæsthesia is carried far enough, totally extinguishes the vital spark. Sometimes too, in the midst of this beautiful, rapid, ebbing, stream of life, when the patient is being rapidly whirled along breaking link after link in the chain of life, dread disease, lying hidden and obscure somewhere in the organism starts up and with rude hand snaps asunder the cords of life.

These are not merely visionary speculations of mine, they are stern practical truths and come home to myself. If I have taken an incorrect position, experience being an excellent teacher and very kindly when properly treated may set me to rights again. Experiment is very valuable and efficacious in teaching us the great truths of Nature, and I shall be proud to avail myself of it on all occasions in which it is justifiable, but I cannot feel justified in experimenting upon my fellow beings when theprobability of a fatal result stares me in the face.

In these few pages I have, I think, illustrated satisfactorily the point which I aimed at, viz.—the necessity for caution in the use of anæsthetics, which is, I regret to say, becoming very much neglected at present. Dentists use them indiscriminately, but more especially is Nitrous Oxide coming into general favor in the Dental profession. I shall certainly exercise caution in their use, letting others if they wish try dangerous experiments. Local anæsthesia scarcely demands a notice in this essay as the general tenor of all my remarks have been in another direction than· the mere action of these agents; still local anæsthetics do have an action on the system and in a few remarks I will pass them by. Their action is quite simple and harmless and if they are half as efficient as they purport to be they are very valuable. By freezing the parts to be operated upon they destroy sensation and render the operation painless. This is the whole modus operandi of their use &c., as is urged for them. I am somewhat suspicious of these agents, I do not regard them with much favor as I have never yet seen a good result follow their use, and I have seen a good many operations performed while under their influence, and never in a single case has the patient professed entire freedom from pain.

